# Toward ‘seeing’ critically: a Bayesian analysis of the impacts of a critical pedagogy

**DOI:** 10.1007/s10459-021-10087-2

**Published:** 2022-01-01

**Authors:** Stella L. Ng, Jeff Crukley, Ryan Brydges, Victoria Boyd, Adam Gavarkovs, Emilia Kangasjarvi, Sarah Wright, Kulamakan Kulasegaram, Farah Friesen, Nicole N. Woods

**Affiliations:** 1 University of Toronto Centre for Interprofessional Education at University Health Network, Toronto Western Hospital, 399 Bathurst St., Nassau Annex (Entrance), Toronto, ON M5T 2S8 Canada; 2 Department of Speech-Language Pathology, University of Toronto, Toronto, ON Canada; 3 Data Science and Statistics, Toronto, ON Canada; 4 Department of Medicine, University of Toronto, Toronto, ON Canada; 5 Institute of Health Policy, Management & Evaluation, Faculty of Medicine, University of Toronto, Toronto, ON Canada; 6 Education, Unity Health Toronto, Toronto, ON Canada; 7 Department of Family and Community Medicine and Wilson Centre, Faculty of Medicine, University of Toronto, Toronto, ON Canada; 8 Wilson Centre, University of Toronto, Toronto, ON Canada

**Keywords:** Critical reflection, Critical pedagogy, Equity, Bayesian, Social responsibility

## Abstract

Critical reflection supports enactment of the social roles of care, like collaboration and advocacy. We require evidence that links critical teaching approaches to future critically reflective practice. We thus asked: does a theory-informed approach to teaching critical reflection influence *what* learners talk about (i.e. topics of discussion) and *how* they talk (i.e. whether they talk in critically reflective ways) during subsequent learning experiences? Pre-clinical students (*n* = 75) were randomized into control and intervention conditions (8 groups each, of up to 5 interprofessional students). Participants completed an online Social Determinants of Health (SDoH) module, followed by either: a SDoH discussion (control) or critically reflective dialogue (intervention). Participants then experienced a common learning session (homecare curriculum and debrief) as outcome assessment, and another similar session one-week later. Blinded coders coded transcripts for *what* (topics) was said and *how* (critically reflective or not). We constructed Bayesian regression models for the probability of meaning units (unique utterances) being coded as particular *what* codes and as critically reflective or not (*how)*. Groups exposed to the intervention were more likely, in a subsequent learning experience, to talk in a critically reflective manner (*how)* (0.096 [0.04, 0.15]) about similar content (no meaningful differences in *what* was said). This difference waned at one-week follow up. We showed experimentally that a particular critical pedagogical approach can make learners’ subsequent talk, ways of seeing, more critically reflective even when talking about similar topics. This study offers the field important new options for studying historically challenging-to-evaluate impacts and supports theoretical assertions about the potential of critical pedagogies.

## Introduction

This paper analyzes the outcomes of one enactment of critical pedagogy, as an approach to teaching for critical reflection, which is a lens for critically reflective practice: a way of being and seeing in practice that orients the practitioner to question assumptions, power relations, and structures, and to change these when they are unhelpful (Ng et al., 2019a, b). This article is important because these approaches to teaching (critical pedagogy) and to practice (critically reflective practice) may offer a way to address an important challenge in health professions education.

How can we prepare professionals to optimally perform the social roles of care? (Mykhalovskiy & Farrell, 2005; Verma et al., 2005; Kumagai, 2014; Ng et al., 2020. These roles include the health advocate, communicator, collaborator, professional, and systems-based practitioner; collectively, they have been variably referred to as humanistic, intrinsic, or social roles (Dyne et al., 2002; Frank & Danoff, 2007; Sherbino et al., 2011; Whitehead et al., 2014). Currently, dominant approaches to prepare practitioners for these social roles include teaching social determinants of health (SDoH) and cultural competence, and using portfolios or similar ‘reflective’ documents. Each of these approaches have encountered considerable critique (Driessen et al., 2005; Kumagai & Lypson, 2009; Metzl & Hansen, 2014; Kuper et al., 2017; Ng et al., 2015a; Sharma et al., 2018).

Critiques include, for example, Sharma et al.’s (2018) discussion of how a SDoH approach can focus learners on race but not racism, poverty but not oppression, homosexuality but not homophobia. In essence, Sharma et al. contend that this focus risks perpetuating rather than transforming the inequities that a SDoH approach espouses to remedy, because it positions SDoH as aspects of individuals rather than consequences of society and systems, and thereby diverts focus onto changing individuals/patients instead of changing systems (Sharma et al., 2018). Approaches to cultural competence similarly risk discounting complex cultural experiences and identities and propagating a reductionist checkbox approach. This oversimplification and overgeneralization of individuality, complexities, and nuances can lead to rote performance of supposed cultural competence that can potentially do harm to patients (Kumagai & Lypson, 2009). Self-reflective portfolios and related self-reflective assignments, which aim to inspire, document, and assess the development of social roles, can instead lead students to feel they must perform inauthentically and are being surveilled (Nelson & Purkis, 2004; Hodges, 2015; Ng et al., 2015a, b; de la Croix & Veen, 2018).

We propose that critical reflection can offer an alternative frame and rise to the challenges highlighted above. This frame and its associated teaching approach, critical pedagogy, are rooted in the critical scholarship of Habermas, Freire, hooks, contemporary education scholars such as Brookfield, Kemmis, and Kincheloe, and health professions education thinkers like Kinsella, Kumagai, and Wear (Habermas, 1971; Brookfield, 2000; Freire, 2000; Kemmis, 2005; Kinsella, 2006; Kumagai & Lypson, 2009; Kumagai, 2014; Hooks, 2015; Ng et al., 2019a, b). Building from the concept of reflection—the active, persistent, and careful questioning of knowledge claims and their sources (Dewey, 1910)—critical reflection focuses less on self and instead turns its gaze to personal and societal assumptions and unhelpful power relations, with the goal of improving how one practices one’s chosen profession (Brookfield, 2000; Freire, 2000). A critically reflective professional adopts a critically reflective way of seeing and being that orients them to practice with a commitment to ethics and justice (Freire, 2000; Kumagai & Lypson, 2009; Ng et al., 2015a, b). For example, professionals with a critically reflective way of seeing may not only understand disability but also notice and change subtle ableism embedded in a rehabilitation plan. They may not only understand how socioeconomic status influences health access but also recognize and aim to mitigate classism embedded in healthcare systems. Past research has demonstrated that critical reflection supports practice that is more compassionate (Rowland & Kuper, 2018), collaborative (Ng et al., 2020), and equitable (Mykhalovskiy & Farrell, 2005), thus providing an opportunity to prepare learners for the social roles of health care.

However, past research has also shown that professionals predominantly learn their critically reflective capabilities through their personal experiences and relationships (Mykhalovskiy & Farrell, 2005; Rowland & Kuper, 2018; Ng et al., 2020). For example, Rowland and Kuper (2018) found that physicians became more critically reflexive, and thus compassionate, after experiencing the patient role firsthand. Ng et al. (2020) found that professionals and patient caregivers attributed their own critically reflective views to personal relationships, past careers, and experiences occurring by happenstance, not to formal education. These forms of personal and experiential learning align with the principles of critical pedagogy, which emphasize learning through experience and engaged participation, and connecting with others’ common humanity through sharing of stories (Kumagai et al., 2009; Halman et al., 2017; Baker et al., 2019, 2020). Nonetheless, the question remains:  how to appropriately integrate critical reflection and pedagogy in more formal health professions education curricula.

Critical reflection scholars, working from social constructionist paradigms, have written about how to teach this way of seeing through critical pedagogy, and their contemporaries have evaluated applications of these approaches using qualitative research designs (Thille et al., 2018; Kumagai & Lypson, 2009). However, in health professions education, an interdisciplinary field traditionally dominated by the biomedical model and experimentalist traditions, proponents of critical reflection and critical pedagogy often face questions of effectiveness. In particular, educators and researchers often expect to see measurable outcomes for educational interventions. However, the purpose of teaching critical reflection is not to enhance the acquisition of content knowledge or the performance of skills in a pre-determined manner; thus, its effects could be missed or misrepresented by traditional written (e.g. multiple-choice questions) and performance-based (e.g. station-based exams) measurements. Instead, we argue that teaching critical reflection influences students’ views about, and capabilities for, practice-based learning and critically reflective practice when they experience indeterminate zones of practice—that is, uncertain, unique, value-conflicted, and dynamic aspects of practice (Schön, 1983; Cheng et al., 2017).

Thus, we propose that studying the teaching of critical reflection experimentally requires alternative approaches to measuring outcomes, in order to maintain paradigmatic compatibility (Tavares et al., 2020) with the origins of critical reflection, while also addressing questions posed by experimentalist models of health professions education. We aimed to fill this gap: to explore, experimentally, the theoretical assertion that teaching critical reflection as a capability can shift health professionals’ ways of seeing in subsequent experiences. We conducted an experiment to address the questions: does teaching critical reflection influence *what* learners talk about (i.e. topic of discussion) and *how they talk* (i.e. whether they talk in critically reflective ways) during a subsequent learning session and debrief? Acknowledging that the passage of time can impact performance, we explored whether any effects changed one week after initial training. We used the way learners talked following a critically reflective dialogic teaching session as our experimental proxy for ways of seeing. This approach to analyzing talk/text to infer a way of seeing is aligned with how scholars have handled other value-laden constructs, such as professional identity (Kalet et al., 2018). Importantly, we did not judge the quality of the reflection but rather whether or not their talk was consistent with a definition of critical reflection that a critical pedagogical approach aimed to teach. We expected that teaching for critical reflection would not have an effect on the *what* of learners’ talk—given critical reflection is less focused on content and more on framing (e.g. noticing not just age but ageism)—and expected it would impact *how* they see and thus talk about the same content (e.g. countering rather than adopting ageist language).

## Methods

### Overview of study design

**Fig. 1:**
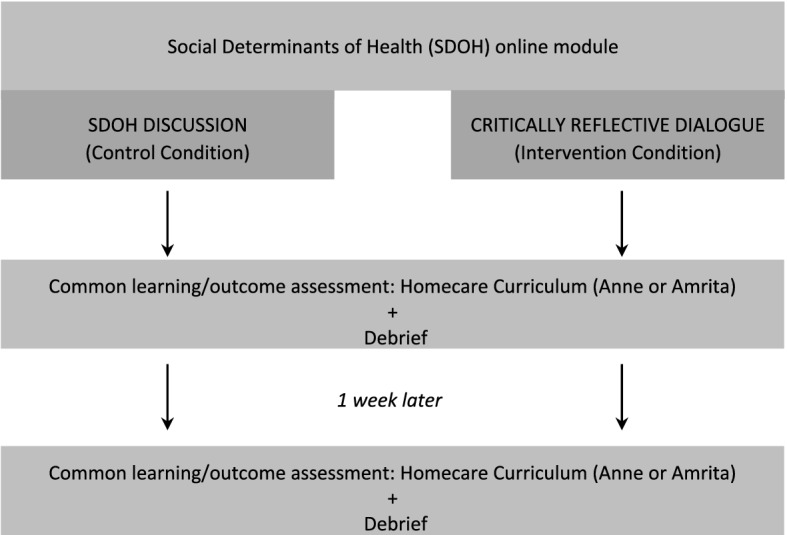
Summary of research design (Mylopoulos & Woods, 2014)

The study was approved by and complied with the University of Toronto Office of Research Ethics and took place in Toronto, Canada. We developed a single teaching and learning session based on theories of critical reflection, reflexivity, and critical pedagogy, and on the teaching materials and scholarship of the first author and colleagues (Kinsella et al., 2012; Ng, 2012; Ng et al., 2015a, b; Phelan & Ng, 2015; Halman et al., 2017; Baker et al., 2018, 2020; Ng et al., 2018, 2020). To test its effectiveness, we employed an established education research design meant to test the transfer of learning to subsequent/future experiences, rather than simply test immediate knowledge acquisition and retention, as outlined in Fig. 1.

This design intended to make visible how participants engage with, or see, a new learning experience following their instructional exposure to the critical pedagogical learning experience. Participants in the control and intervention conditions completed an online module about SDoH, followed by different instructional exposures: either a critically reflective dialogue or further SDoH discussion. Then, participants in both conditions experienced two common learning sessions that served as our outcome assessment, one immediately after the instructional exposure and another one-week after the initial training. These two common experiences enabled measurement of the outcomes of interest.

### Teaching materials

The teaching materials used in this study are summarized in Table 1. They included an online module on SDoH (for both control and intervention conditions) and guides for the follow-up SDoH discussion (control) and for the critically reflective dialogue (intervention). An online homecare curriculum and a debriefing guide to follow the homecare curriculum served as the common learning resources that generated the outcome measures.

**Table 1:** Study materials

Session	Condition that received the session	Source and details
Social determinants of health (SDoH) online module	Control and Intervention	Available for free from the Canadian Nurses Association (http://cnaaiic.ca/html/en/Social-Determinants-of-Health_e-Learning-Course/module1/story.html)
SDoH discussion	Control	Custom-designed discussion guide by principal investigator—see "Appendix 1"
Critically reflective dialogue	Intervention	Custom-designed critical reflection dialogue session by principal investigator—see "Appendix 2" for slides. Uses contrasting cases and a thought-provoking image followed by guiding questions to spark a critically reflective dialogue. The session design was informed by theories of critical reflection and principles of critical pedagogy
Homecare curriculum	Control and intervention	The CACE Homecare Curriculum: An interprofessional, interactive, and multi-media online curriculum developed by the first and last author, focused on older adult homecare Three cases/modules exist—Amrita (with a content focus on dementia), Anne (with a content focus on delirium), and Alexei (with content focus on depression). For this study, only the Amrita and Anne cases were used (http://www.capelearning.ca)
Homecare curriculum debrief	Control and intervention	Adapted from Cheng et al.’s PEARLS debriefing approach See "Appendix 3"

### Participants

An interprofessional group of students (n = 75) were recruited from: the first two years of the University of Toronto MD program (n = 31), a fourth-year undergraduate pre-clinical service-learning course with students of mixed degree backgrounds (n = 18), the first year of master’s level occupational therapy (n = 6), physical therapy (n = 6) and speech-language pathology (n = 10), and one student from dentistry, pharmacy, physiotherapy assistant, and radiation therapy programs (n = 4). We chose pre-clinical and early year students to minimize the amount of formal health professions education and clinical practice exposure they would have had.

Participants were assigned to sixteen groups of five, ensuring a multiprofessional complement of students per group to enable interprofessional learning. Eight groups were randomly assigned to the control condition and eight to the intervention condition. We began the study with 80 participants but five participants—2 from the control condition and 3 from the intervention condition—dropped out prior to participation due to factors external to the study (e.g. weather conditions).

### Procedures

A research coordinator consented students and managed administrative elements of the data collection sessions. Both control and intervention participants completed a short SDoH curriculum online module. Each group of five participants completed this SDoH online module in a computer lab, each at their own pace using headphones for privacy, with a research coordinator present to ensure no distractions.

In their same groups of five, learning then diverged as participants proceeded to either a SDoH discussion (control) or critically reflective dialogue session (intervention). All sessions were completed in a small student lounge, with chairs set up in a circle, with the discussion/dialogue facilitator seated along with participants. These details align with a critical pedagogy approach in which the teacher-learner hierarchy is minimized and a learning climate conducive to openly challenging assumptions is emphasized. The SDoH session used a semi-structured facilitation guide (“Appendix 1”). A television screen was connected to a laptop to project supporting slides for the critical reflection session only (“Appendix 2”). Participants were provided with food and beverage during these sessions. Both the control and intervention conditions were one-hour in duration. Sessions were audio-recorded and later transcribed verbatim. The facilitators both held master’s degrees and were trained to run their sessions by the first author. To minimize the confounding variable/factor of individual facilitation styles, the facilitators for the SDoH discussion and critically reflective dialogue session switched roles at the halfway point of data collection. We also included facilitator as a factor in the analysis to account for any potential effects.

After completing the SDoH discussion or critically reflective dialogue sessions, participants were given a brief 10-min break before reconvening within the same groups in a small conference room. The setup included a table for participants and facilitator, and television screen to display the learning resource, the CACE Homecare Curriculum (http://www.capelearning.ca). A third facilitator ran the homecare curriculum and debriefs. Our aim was not to determine whether the participants learned about homecare or applied SDoH to the homecare context. Instead, we used the homecare curriculum followed by a debrief to uncover whether the critically reflective dialogue session, relative to the SDoH discussion, influenced *what* topics participants talked about during the curriculum and debrief, as well as *how* they talked during these experiences (in a critically reflective manner, or not). Participants were instructed to act as though they were directly involved in the patient case module. Each group completed one 35-min module from the curriculum—either “Amrita” (dementia-focused) or “Anne” (delirium-focused)—with the facilitator’s guidance. The sessions were audio-recorded and transcribed verbatim.

The facilitator then guided a 30-min debriefing with participants, within their groups, immediately after completing the homecare curriculum. The facilitator was trained to use the Promoting Excellence and Reflective Learning in Simulation (PEARLS)-informed (Eppich & Cheng, 2015) debriefing script (see "Appendix 3"). The debrief prompted participants to discuss their experience of the homecare curriculum, thoughts on the older adults’ situations and needs, and key lessons learned. The debriefing was audio-recorded and transcribed verbatim. The homecare/debrief facilitator also held a master’s degree, was trained by the first author, and remained blinded to each group’s condition assignment.

One week later, participants were brought back in their groups to complete the follow-up homecare curriculum module plus debrief to determine if any effects identified in the previous session persisted or changed. All participants completed another patient case module (if they received the Amrita module, they now received Anne, and vice versa) followed by a similarly structured debrief (“Appendix 3”). The sessions and debriefs were audio-recorded and transcribed.

### Coding

Analysis was performed on each group of five interprofessional learners’ transcripts from the control (SDoH discussion) and intervention (critically reflective dialogue) sessions, as well as the homecare curriculum sessions and debriefs immediately post-instruction and at follow-up.

Before coding, meaning units were created, to ensure consistency in the amount and type of text to which *what* and *how* codes were subsequently applied, and that our two coders coded the same segments of text. Meaning units were created as follows. Within each transcript, every unique utterance by a participant (the boundaries of a unique utterance were determined by change in speaker) was labelled as a meaning unit, unless it met the exclusion criteria of: facilitator comments, neutral affirmations (mhmm, etc.), responses to homecare module quiz questions, responses about the quality of the module with no additional comment about content (e.g. “This module is fun”), responses to questions about participants’ program/year, or clarifying questions (facilitator asks a question and participant asks for clarification, and statements that added no meaning). Every meaning unit was then ready to be coded with at least one *what* code and one *how* code.

To create the *what* coding framework, two researchers VB (co-author) and the first author, initially coded transcripts inductively to arrive at an agreed upon set of eight descriptive codes plus a “no code” code and associated descriptions for each code. They iteratively defined codes until they could be applied strictly to name the topic of meaning units, without further changes. While there were sub-codes used to assist the coding process, only the higher-level codes were used in our statistical analyses.

For the *how* coding framework, the following definition was used to code data as critically reflective, or not. Meaning units coded as critically reflective were statements that: move beyond a dominant discourse (e.g. discuss a social model as opposed to medical model of disability), question individual or societal assumptions/beliefs (e.g. a clinician’s belief that they have authority over what a school does for a child with disability), demonstrate awareness of the broader system and how one is situated (e.g. recognize that personal support workers may lack resources and training opportunities relative to a regulated health professionals), question or challenge structures (e.g. question whether current funding approaches for homecare are limiting possible practices), and resist harmful practices (e.g. speak up if noticing something concerning) (Ng et al., 2019a, b). Meaning units coded as not critically reflective were: neutral descriptions, narrow views (e.g. illustrated through stigmatizing language), rote following/description of procedures or steps, blaming or patronizing the patient/caregiver/health worker.

Two blinded coders (VB, co-author and LN, acknowledged) were trained to apply these codes to the transcripts. We calculated the inter-rater agreement on eight transcripts to ensure the coders applied the codes consistently. For *what* codes, raw rater agreement was 95.9%; for *how* codes it was 82.1%. We determined that this was sufficient to allow the two coders to proceed independently, and subsequent transcripts were only coded by one coder each.

### Statistical analysis

We conducted our analysis in accordance with the aims of this study: to investigate whether teaching for critical reflection influenced *what* students talked about during a future learning experience and *how* they talked about it. Thus, we constructed two regression models, one to model the presence of a *what* code in each meaning unit and one to model the presence of a *how* code in each meaning unit.

Paradigmatically aligned measurement and analyses were crucial to this study. We constructed, tested, and selected our models under a Bayesian framework. The following are our justifications. Frequentist inference addresses the question of how probable a set of observed data would be if there were no effect. We were specifically interested in addressing the question of how likely a code would be present in a meaning unit as a result of our intervention, which is an inherently Bayesian question. Bayesian inference directly quantifies uncertainty. It provides estimates of the probability of parameter estimates, their differences, and the uncertainty in those estimates and thus any conclusions drawn. Bayesian inference treats the gathered data as ‘fixed’ and models and their parameters as ‘varying’ attempts to explain the observed (‘fixed’) data, while quantifying the model and parameter uncertainty. Some in medical education have compared Bayesian statistics to constructivist grounded theory for the way in which it positions models as imperfect best attempts at representing the story of the data, with analyses informed by prior knowledge (Young et al., 2020). For details on Bayesian statistics, particularly details on the posterior distribution, we recommend McElreath (2020). All models were constructed using the Stan programming language (Carpenter et al., 2017) through the rstan (Stan Development Team, 2020) and brms (Burkner, 2017, 2018) packages in R statistical computing software (R Core Team, 2019).

We modelled predictive probabilities of *what* codes with a hierarchical multinomial regression model. Meaning units were categorized as containing any or all of nine codes, based on our inductive coding framework: Building on prior knowledge, CanMEDS roles, caregiver, professional expertise, patient, patient-psychosocial, recommendations for practice, social determinants of health, or no code for meaning units that remained in the codable set despite lacking relevance to any of the eight main codes. The definitions of these codes are included in the codebook within "Appendix 4". Population-level effects in this model included condition (control or intervention), session (initial instruction, initial homecare, initial debrief, follow-up homecare, follow-up debrief), and facilitator (Facilitator 1 or Facilitator 2). We also included interaction terms for: code*condition, code*session, condition*session, and facilitator*session. SDoH discussion group (for control participants) or critically reflective dialogue group (for intervention participants) was entered as a varying effect to adjust for clustering of meaning units within each five-member group. The presence of “no code,” at the Instruction Session, in the Control Condition, with Facilitator One was used as the reference case. We used mildly informative priors on the regression coefficients: α_i_ ~ N(0,1), β_i_ ~ N(0,1), σ_i_ ~ cauchy(0, *e*^1^).

We used Pareto smoothed importance sampling leave-one-out cross-validation (PSIS-LOO) (Vehtari et al., 2016) to evaluate and compare the fit of our full (hierarchical model) to an “empty” (intercept-only) model, and a non-hierarchical model (with the same population-level effects) to the data. We found our full model to be a significantly better fit to the data relative to both (i) the intercept-only model, with a favorable difference in PSIS-LOO expected log predictive density (ELPD) of 1599.8 (with a standard error of 42.2), and (ii) the non-hierarchical model with an ELPD of 93.9 (with a standard error of 13.3).

To model the predictive probabilities of the *how* codes, we constructed a hierarchical binary logistic regression model. Meaning units were categorized binomially as either present or absent of critical reflection. Population-level effects in this model included condition (control or intervention), session (instruction, initial homecare, initial debrief, follow-up homecare, follow-up debrief), and facilitator (Facilitator 1 or Facilitator 2). We also included interaction terms for: condition*session and facilitator*session. As in the *what* model, SDoH discussion group (for control participants) or critically reflective dialogue group (for intervention participants) was entered as a varying effect to adjust for clustering of meaning units within five-member groups. The Instruction Session, in the Control condition, with Facilitator One, was used as the reference case. We used mildly informative priors on the regression coefficients: α_i_ ~ N(0,1), β_i_ ~ N(0,1), σ_i_ ~ cauchy(0, *e*^1^).

As conducted with our *what* model, we used PSIS-LOO to evaluate and compare the fit of our full (hierarchical model) to an “empty” (intercept-only) model, and a non-hierarchical model (with the same population-level effects) to the data. We found our full model to be a significantly better fit to the data relative to both (i) the intercept-only model with a favorable difference in PSIS-LOO ELPD of 172.6 (with a standard error of 15.5), and (ii) the nonhierarchical model with an ELPD of 18.0 (with a standard error of 6.1).

To evaluate the effect of our intervention, we calculated the distribution of differences in posterior predicted probabilities of codes being present in each Session Type and then determined if the 89% highest density credible intervals of these predicted difference distributions included zero where the inclusion of zero was taken as an indication of no difference between Conditions. The credible interval is the range of the posterior distribution containing the specified proportion (i.e. 89%) of the parameters of interest (i.e., the differences in posterior predicted probabilities) such that one could say: “given the observed data, the effect has an 89% chance of falling in this range” as opposed to a less intuitive frequentist confidence interval, which would be interpretable as “there is an 89% probability that when computing a confidence interval from data of this sort, the effect falls within this range” (Makowski et al., 2019). The 89% credible interval is recommended by a number of leading Bayesian statistics thinkers for its *computational stability* relative to 95% intervals (Kruschke, 2014). While 90% was also proposed for this same reason, McElreath (2014, 2020) suggested that 89% makes potentially more sense because 89 is “the highest prime number that does not exceed the already unstable 95% threshold.”(Makowski et al., 2019a, b). Because Bayesian analyses yield probability distributions of parameter values (e.g., regression coefficients) calculated directly from observed data, they do not require calculation of *p* values or their associated confidence intervals. Rather, uncertainty is quantified directly from the calculated probability distributions of parameters (Kruschke & Liddell, 2018).

## Results

### What learners talked about

**Fig. 2:**
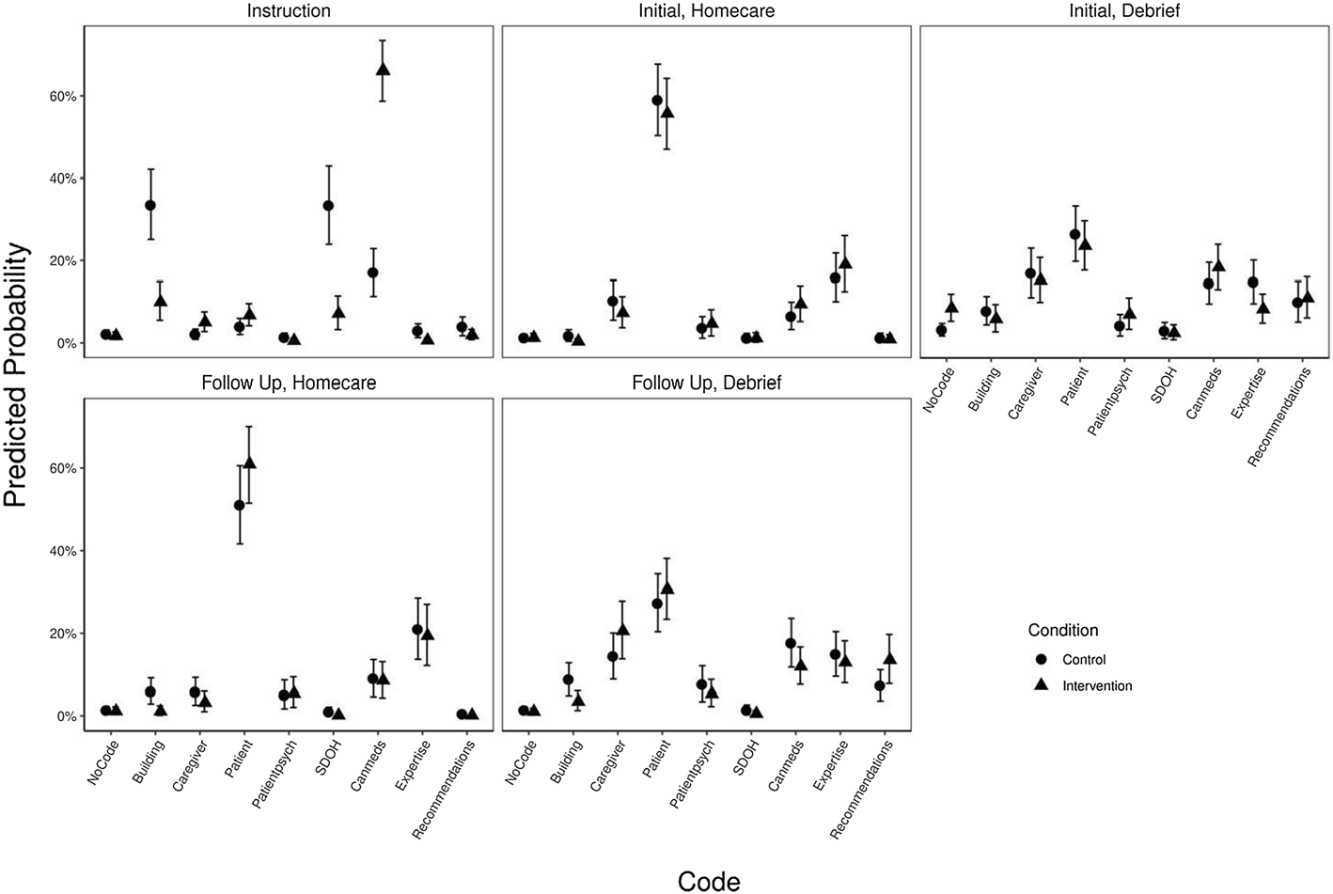
Mean predictive probabilities by session type and condition for “what” codes

A summary of our final hierarchical model for *what* codes is presented in Fig. 2 and a regression summary table of our “empty” (intercept-only) model, a non-hierarchical model, and our final hierarchical model for *what* codes is included in "Appendix 5".

Figure 2 shows mean posterior predicted probabilities together with the respective 89% highest density credible intervals of *what* codes by session type and condition.

We see a higher probability of “CanMeds” being coded for meaning units deriving from the critically reflective dialogue instruction sessions (0.64 [0.57, 0.71]) than the SDoH instruction sessions (0.17 [0.11, 0.23]), and a higher probability of “building on prior knowledge” and “SDoH” being coded during the SDoH instruction sessions (0.33 [0.24, 0.44] and 0.35 [0.24, 0.46], respectively) than in the critically reflective dialogue instruction sessions (0.1 [0.06, 0.15] and 0.07 [0.03, 0.11], respectively). Otherwise, the probabilities of codes being applied to a meaning unit were virtually equivalent between conditions for our *what* codes.

**Fig. 3:**
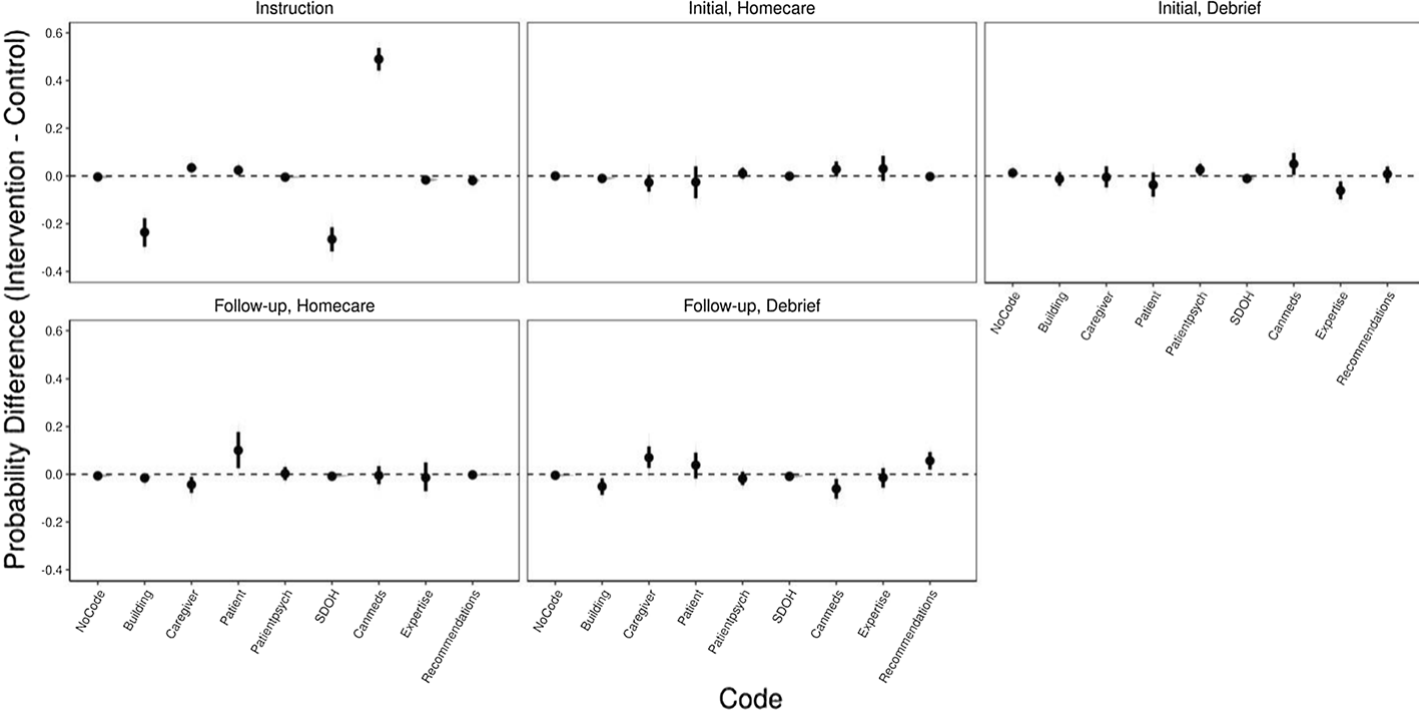
Posterior predicted difference distributions by session type for “what” codes

Figure 3 depicts the posterior predictive difference distributions for each code in each Session Type between SDoH and critically reflective dialogue groups. Points represent mean probability differences; bars represent the 89% highest density credible interval surrounding the means; shaded regions indicate the distribution of posterior probability differences, and the dashed lined marks zero.

As depicted in Fig. 3, the probability of “building on prior knowledge” and “SDoH” being coded were more likely for the SDoH instruction sessions than in the critically reflective dialogue instruction sessions (differences 0.24 [0.31, 0.16] and 0.26 [0.33, 0.2], respectively). The probability of “CanMeds” being coded was more likely in the critically reflective dialogue instruction sessions than in the SDoH instruction sessions (difference of 0.49 [0.43, 0.55]).

Overall, these results show that during the common learning conditions (i.e. both initial and follow-up) serving as our outcome assessment, no *what* codes were more, or less, probable among meaning units deriving from the intervention versus control conditions.

### How learners talked

**Fig. 4:**
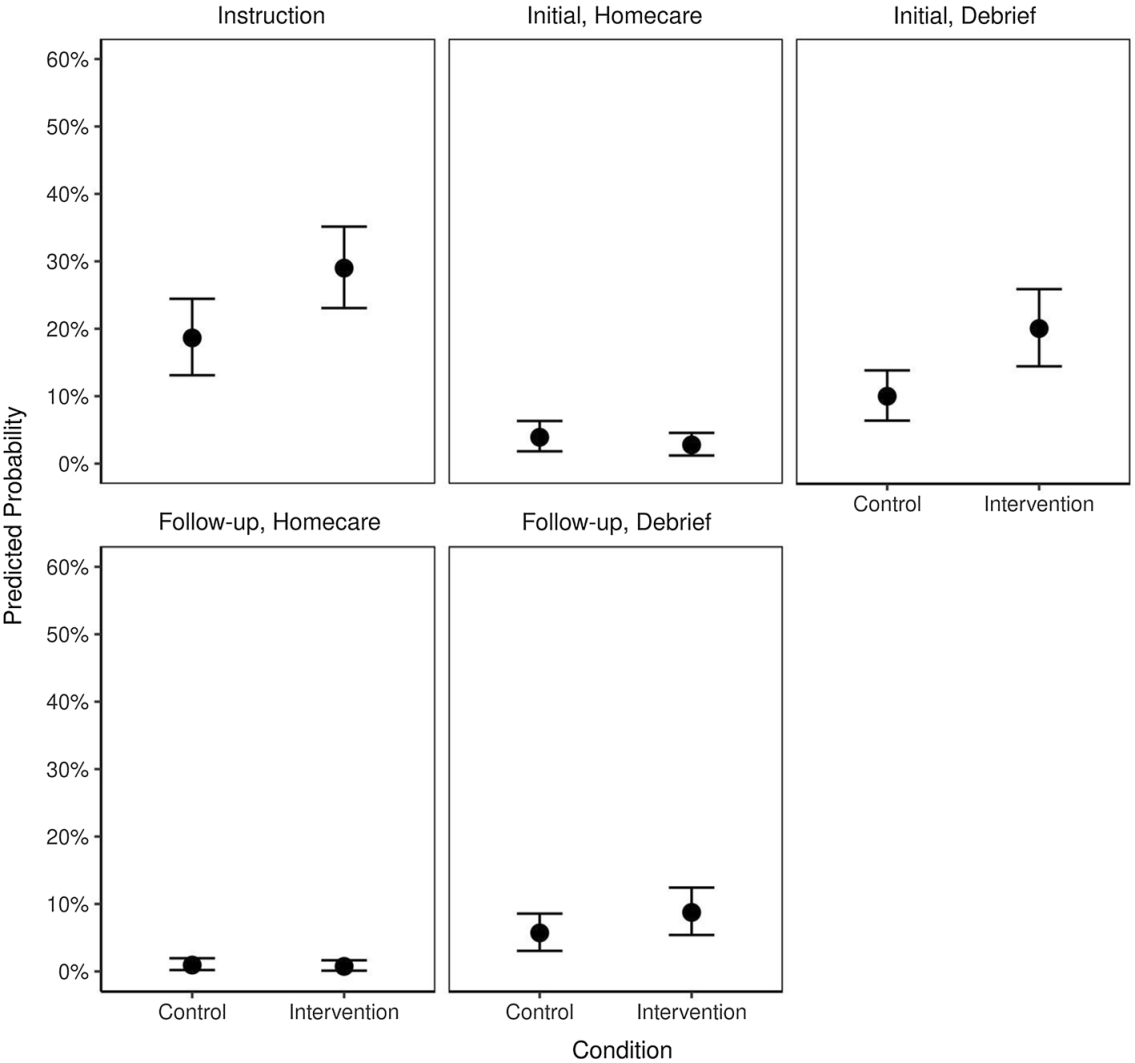
Mean predictive probabilities for meaning units coded as critically reflective by session type and condition

A summary of the final model for *how* participants spoke (critically reflective or not) is presented in Fig. 4. The regression summary table of our “empty” (intercept-only) model, a non-hierarchical model, and our final hierarchical model for *how* codes is included in "Appendix 5".

Overall, there was a 10% difference between the probability of a meaning unit being coded as “critically reflective” between control and intervention conditions at initial debrief, though the probability of any meaning unit being coded as such was low across all sessions (instruction, initial and followup homecare and debrief) (Table 2).

**Table 2:** Probabilities of critically reflective codes per meaning unit within condition and session type

Condition	Session type	Estimate [89% CI]
Control	Instruction	0.18 [0.12, 0.25]
Control	Initial, Homecare	0.04 [0.01, 0.07]
Control	Initial, Debrief	0.1 [0.06, 0.15]
Control	Follow Up, Homecare	0.01 [0, 0.02]
Control	Follow Up, Debrief	0.06 [0.03, 0.09]
Intervention	Instruction	0.3 [0.24, 0.37]
Intervention	Initial, Homecare	0.03 [0.01, 0.05]
Intervention	Initial, Debrief	0.2 [0.13, 0.27]
Intervention	Follow Up, Homecare	0.01 [0, 0.02]
Intervention	Follow Up, Debrief	0.09 [0.05, 0.13]

**Fig. 5:**
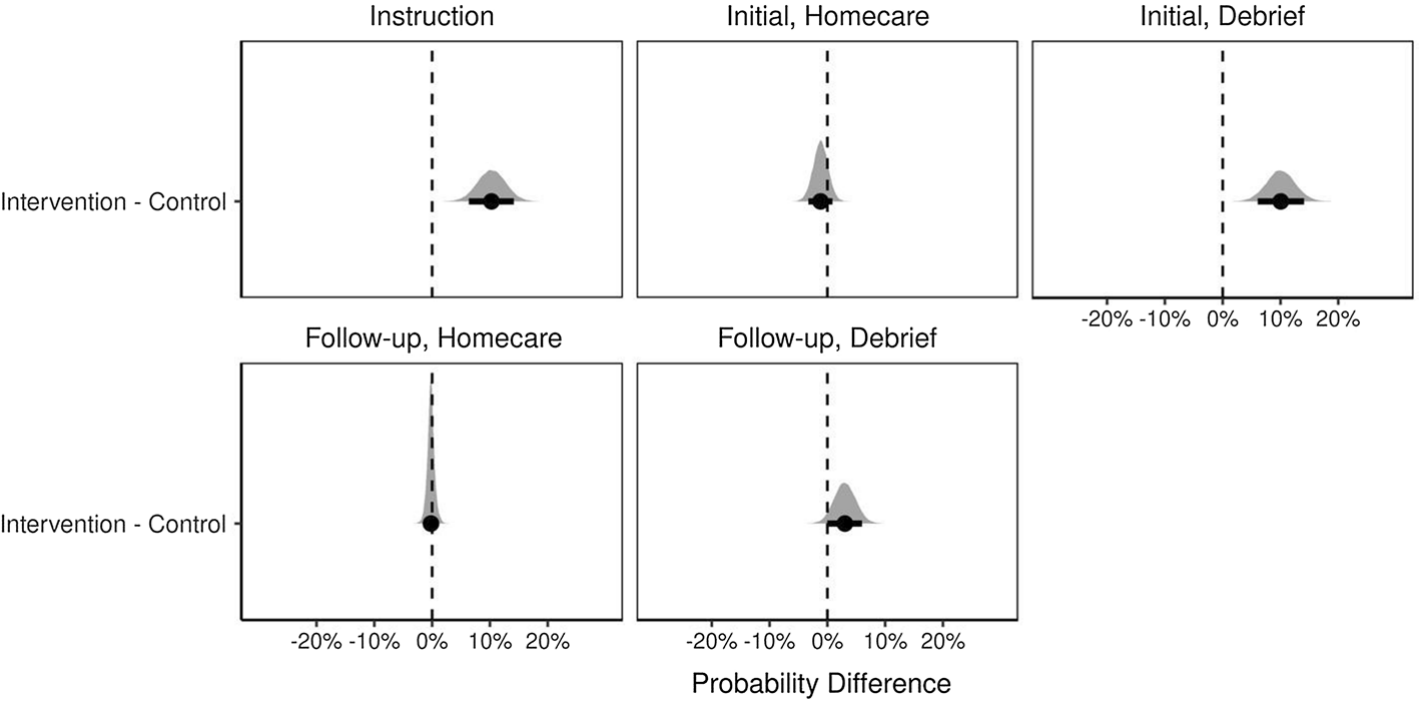
Posterior predictive difference distributions for meaning units coded as critically reflective by session type

Figure 5 depicts the posterior predictive difference distributions for meaning units being coded as critically reflective in each Session Type between SDoH and critically reflective dialogue groups. Points represent mean probability differences; bars represent the 89% highest density credible interval surrounding the means; shaded regions indicate the distribution of posterior probability differences, and the dashed lined marks zero.

The probability of a meaning unit being coded as “critically reflective” was higher for the intervention condition at instruction (0.112 [0.05, 0.17]) and initial debrief (0.096 [0.04, 0.15]).

## Discussion

We asked whether a critically reflective dialogue session would impact *what* learners talked about and *how* they talked. Answering this question, experimentally, is important to conversations about teaching for the social aspects of healthcare. As anticipated, while the instructional conditions focused on different content, the probability of specific content being discussed during the homecare and debrief outcome measurement was similar between participants exposed to the intervention versus control condition. By contrast, and as hypothesized, we did observe an impact on *how* participants spoke, with those exposed to the critically reflective dialogue sessions tending to speak in more critically reflective ways in their subsequent learning experience. This impact was seen even though learners moved to a novel context/content area for the future learning activity (from a pediatric context to an older adult context). This impact during the initial common learning session did not persist well over time, as the difference in probability was markedly reduced at the one-week follow up session. Overall, this study showed that we could demonstrate the impact of teaching for critical reflection on learners’ subsequent ways of seeing, utilizing novel and paradigmatically congruent analysis methods; albeit the observed probability differences between conditions were small.

### Interpreting the results and outcomes

In terms of *what* learners talked about, it makes sense that the control instruction session led to more talk about SDoH and the intervention instruction session to more talk about CanMEDS. The SDoH session specifically asked learners to reflect on SDoH. The critical reflection sessions did not explicitly focus on CanMEDS; however, critical reflection affords a focus on these roles. The cases used to spark dialogue during the critical reflection sessions focused on issues that would invoke contemplation about approaches to collaboration, advocacy, and professionalism. Beyond initial instruction, however, there was no effect of condition on *what* learners talked about during their future homecare curriculum or debrief. This finding suggests that teaching critical reflection in the dialogic manner we used neither adds to nor detracts from learners’ topical content focus as they move forward in their learning.

Of most interest to us is *how* learners talked—specifically, our finding that the learners exposed to critically reflective dialogue went on to produce more critically reflective meaning units during their future learning—when debriefed about their homecare curriculum experience. While the difference in probability between control and intervention groups is low at first glance (10%), we interpret this difference as meaningful. Overall, we would expect the potential for any utterance to be critically reflective, during the homecare curriculum and debrief, to be low for two reasons. First, the intervention produced minimal exposure to critical pedagogy and second, over the course of the homecare curriculum and debrief participants would make many descriptive statements that would by nature have low potential or need to be critically reflective. Indeed, the probability for a meaning unit to be critically reflective even in the intervention condition was only 20%, which is low and expected for the aforementioned reasons. Thus, a small difference is meaningful to this initial experimental study that explores the outcomes of teaching for critical reflection. Credible intervals and the degree of belief, in the form of uncertainty estimates, are generally the focus of Bayesian approaches rather than the point estimates and binary decisions (i.e. reject the null) used in frequentist approaches. In our study, our measure of central tendency (median estimates) of differences was close to zero with narrow credible intervals in almost all *what* codes. Of import to our main research question, for our *how* codes, zero was not a probable estimate*.* We can be confident in our conclusions because the models indicated the data moved the posterior distributions away from the priors. Taken together, this information indicates sufficient data from which to draw meaningful inference (Kruschke & Liddell, 2018; McElreath, 2020).

As in all studies, our interpretations must be constrained according to our design and its limitations. Our sample size was relatively small and heterogenous, although all were university students intending to practice as health professionals in the future. In aiming to balance paradigmatic alignment (Creswell, 2003; Baker et al., 2020; Tavares et al., 2020) with experimental design principles, we may have imposed some limitations on our experimental approach. One such design choice was our group-level focus. Our learning conditions occurred in groups and our analyses did not code utterances per individual. We justified this because we wanted to elicit conversations in an interprofessional group learning / simulated practice situation, during which students were unaware that we would be coding their talk for critical reflection; we expected this would prevent them from performing to our measure. A study by Thille et al. (2018) evaluating a dialogic approach to foster critical reflexivity also analyzed group conversations, preserving group dynamics purposefully. Likewise, our group debriefs enabled us to obtain an aligned and authentic sense of learners’ ways of seeing as they talked amongst their groups. However, while these choices were paradigmatically justified for critical reflection and pedagogy, our focus on the group as a dialogic unit meant that we were unable to see whether individual learners consistently enacted critically reflective ways of seeing.

While many scholars—including some on our author team—have argued that assessing reflection is fraught (Sumsion & Fleet, 1996; Hodges, 2015; Ng et al., 2015a), this study makes an important contribution by demonstrating that two raters can reliably code whether a statement is critically reflective or not. This study was about outcome of teaching, not about assessment of individuals. We looked at presence or absence of critical reflection to determine whether a teaching approach had its intended effect; we did not assess the quality of critical reflection. We continue to caution strongly against judging the quality of an individual’s reflective thought; as others note, doing so can take on a tone of surveilling learners’ personal and political beliefs (Nelson & Purkis, 2004; Hodges, 2015). Further, assessing reflection may take away from its authenticity, making it overly prescriptive (Hodges, 2015; Ng et al., 2015a; de la Croix & Veen, 2018). Arguably, *critical* reflection is more readily identifiable and assessable than other forms of reflection because the critical lens derives from a body of critical theory (Ng et al., 2019b). Thus, the forms of knowledge one would expect to observe are pre-defined and can be identified. That said, we still argue that assessing the *quality* of critical reflection as opposed to its *presence* may be problematic for both psychometric reasons and reasons of paradigmatic misalignment (Sumsion & Fleet, 1996; Koole et al., 2011; Moniz et al., 2015; Ng et al., 2015a, b; Grierson et al., 2020).

### Educational design

When interpreting our main findings, it is important to draw attention to three main elements of educational design. First, our pedagogical approach to the critically reflective session was carefully and deeply informed by theories of how to foster critical reflection (Ng, 2012, Ng et al., 2015a, 2019a). Rather than teaching learners theory about critical reflection, these sessions aimed to equip them to disrupt dominant assumptions, to address the power differentials in the room, to ask questions rather than aim to identify solutions, and to envision ways forward beyond the status quo. They experienced real case examples from past practice-based research (Ng et al., 2015b; Phelan & Ng, 2015).

Second, the learning environment setup was purposeful and aligned with critical pedagogical principles (Greene, 1986; Hooks, 1994; Freire, 2000; Halman et al., 2017;). Facilitators sat in a circle with participants to reduce the sense of a facilitator-participant power differential, the presence of food was intended to foster comfort and informality, the spaces and lighting were chosen to help construct an environment conducive to dialogue. These features of the “instruction” would be considered important for fostering dialogue as they promote comfort and connection amongst learners and the facilitator. These environmental features were the same for both the control and intervention conditions; future research could explore the influence of such factors in learning. We believed that attending to these features may be important to overcome the challenges in setting up critical pedagogies against a dominant backdrop of objectivity and certainty in the health professions (Kuper et al., 2017; Ng et al., 2019a, b; Whitehead et al., 2011) but this belief would need to be explored in future studies. Along these lines, calling the critically reflective dialogue session “instruction” demonstrates the dominance of objectivist paradigms and is incongruent with principles of critical pedagogy. Notably, we use “instruction” to be consistent with our education research design; however in critical pedagogy, there is less of an emphasis on a single instructor or instruction per se, as learners bring their own valid experiences and knowledge worth sharing, to the table, alongside the facilitator’s experience and knowledge (Hooks, 1994; Freire, 2000; Halman et al., 2017).

Third, we allotted a limited amount of time/exposure to critically reflective learning, for study feasibility and to represent the reality in health professions education. Many lament this “tacking on” or “squeezing in” of one-off, brief educational experiences deriving from alternative traditions (Ousager & Johannessen, 2010; Diachun et al., 2014; Charise, 2017). Indeed to see the effect of critical pedagogy persist over time, critical approaches to education would need to be embedded more consistently, comprehensively, and longitudinally in a training program (Ng et al., 2020). A potential consequence of this increased intensity could be an increased probability of learners’ utterances being critically reflective.

One promising approach to bridge critical pedagogy with cognitivist education paradigms (which emphasizes knowledge acquisition as opposed to ways of seeing and being [Baker et al., 2020]) may lie in our debriefing script based on Cheng’s PEARLS framework (Eppich & Cheng, 2015). That there was no effect of condition on how learners talked during the homecare curriculum itself was unsurprising as it was a structured curriculum. But during the debrief, in which more open and critical reflection prompting questions were posed, the intervention did have its expected effect. Questions like “what were your thoughts about the family caregiver” could in and of themselves trigger critical reflection. For example, this question may trigger insights or queries about whether the health and social care systems adequately support such caregivers. The difference between learners who were exposed to the intervention versus those who were not suggests that the intervention supports learners to take greater advantage of such debriefing prompts. It is possible that the PEARLS approach to debriefing is a form of continued critical reflection teaching. However, the reduced impact at the one-week follow up debrief suggests that continued debriefing alone is insufficient to sustain the effects of the critical reflection teaching. Future research could delve more deeply into the most effective way to sustain critical reflection throughout and beyond formal health professions training.

Ultimately, we believe this study offers a much-needed bridge between critical pedagogy aiming to foster critical reflection, and experimentalist approaches to studying educational interventions. In the interdisciplinary field of health professions education, paradigmatic diversity offers challenges and opportunities (Young et al., 2020). We sought to reconcile a difference that caused much collegial debate amongst members of our interdisciplinary team. Experimentalists argued that claims about the effects of teaching for critical reflection were under-substantiated given a paucity of experimental evidence. The critical pedagogues argued that such forms of evidence would relegate authentic, embodied reflective capabilities to inauthentic and overly prescriptive caricatures. Through interdisciplinary debate and dialogue, our team developed an approach to advancing knowledge on the teaching of critical reflection that satisfied both perspectives. What the field does with this bridge remains to be seen; it may be prudent to proceed with commitment to criticality and dialogue as both our research reported in this article, and the pedagogy within it, demonstrated.

## Appendix 1: SDoH discussion guide

- What did you think of that learning module?
- Did anything surprise you?
- Did anything seem confusing to you?
- Have you had personal experiences in which the social determinants of health have influenced:
  - Your well-being
  - The well-being of a close friend or family member?
    - How did you feel?
    - How did you handle this?
    - Would you do things differently in the future?
- As a future health professional, what will you do to help address the social determinants of health?
- Do you have anything else to add?

## Appendix 4: Codebook for “what” codes

**Table:**

Code	Subcodes	Description
Building on prior knowledge-problem solving-integration		Integrating personal experience/knowledge—Integrating past experience/knowledge—Integrating formal curricular experience/knowledge
Caregiver		
	Caregiver Competence	Knowledge, capacity to care
	Caregiver Condition	e.g. Physical or emotional state, burnout, health
	Caregiver needsrecommendations	e.g. Additional support—various explanations as to why, physical mobility
	Safety concerns	
Patient		
	Cognitive State-Memory loss	
	Emotional state-Mental health	
	Patient Behaviour	
	Safety concerns	
	Signs, symptoms, medication	
	Patient needs	
Patient psycho-social factors		
Social Determinants of Health consistent with CNA SDoH curriculum used in study		
	Access to education	A good education & culturally appropriate school environment leads to better health. Cost is a significant barrier to continuing education after high school
	Access to healthy food	Eating poorly can have a number of negative health effects (e.g. increased food allergies, diabetes, hypertension). Household food insecurity is linked to poor general health (e.g. chronic conditions, depression). Food security appears to be on the rise in Canada with increased food bank use
	Access to Housing	Good housing (stable & safe) > better health. Poor housing (e.g. heating deficiency, overcrowding, vermin) > worsening health. Home inadequacies may increase exposure to lead, poor heating, etc. Homelessness is the most severe form of housing insecurity. Homelessness has the greatest impacts, including early death
	Built and natural environment	Where you live can affect your health. Rural living can mean less pollution—but also less access to health-care services. Urban living
		can mean better access to services—but greater exposure to pollution
	Conditions of early childhood	Critical stages of physical, mental, and emotional development. Fundamental in creating a healthy foundation for adult health. Keys to positive long-term effects: Health attachments, stimulation, access to quality child care, access to activities (e.g. dance)
	Employment and working conditions	A stressful job can contribute to other physical & psychological problems. Stress may be increased by SDoH such as lower incomes & less education. BUT good workplace conditions support well-being
	Income	Individuals, families, and communities need sufficient income. Poorer people experience more illness and shorter lives than the rich
	Social inclusion	Ability to participate in social & cultural activities, including paid work and civil affairs. Ability to enjoy social goods such as health care, education, and language services. Exclusion commonly experienced by vulnerable groups (e.g. recent immigrants, elderly, people with disabilities, LGBTQ, Indigenous b/c more limited access to resources). Unemployment among recent immigrants twice that of Canadian-born workers
CanMeds roles, per the Royal College of Physicians & Surgeons of Canada		
	Advocacy	Respond to an individual patient’s health needs by advocating with the patient within and beyond the clinical environment
	Collaboration	1. Work effectively other colleagues in the health care professions 2. Work with other colleagues in the health care professions to promote understanding, manage differences, and resolve conflicts. 3. Hand over the care of a patient to another health care professional to facilitate continuity of safe patient care
	Communication	1. Establish professional therapeutic relationships with patients and their families
2. Elicit and synthesize accurate and relevant information, incorporating the perspectives of patients and their families. 3. Share health care information and plans with patients and their families 4. Engage		
		patients and their families in developing plans that reflect the patient’s health care needs and goals 5. Document and share written and electronic information about the medical encounter to optimize clinical dec
	Medical expertise	1. Practise medicine within their defined scope of practice and expertise 2. Perform a patient-centred clinical assessment and establish a management plan 3. Plan and perform procedures and therapies for the purpose of assessment and/or management 4. Establish plans for ongoing care and, when appropriate, timely consultation 5. Actively contribute, as an individual and as a member of a team providing care, to the continuous improvement of health care quality and patient safety
	Professionalism	1. Demonstrate a commitment to patients by applying best practices and adhering to high ethical standards 2. Demonstrate a commitment to society by recognizing and responding to societal expectations in health care 3. Demonstrate a commitment to the profession by adhering to standards and participating in physician-led regulation 4. Demonstrate a commitment to physician health and well-being to foster optimal patient care
Professional Expertise		For PSWs, something like managing responsive behaviours would count as their expertise as this is a big focus for their work, whereas something like medical knowledge is actually not expected of them
Recommendations for practice		

## Appendix 5: Regression Summary Tables

**Table:** What—regression summary table

Predictors	Intercept-only model Odds ratios (89% CI)	Non-hierarchical model Odds ratios (89% CI)	Hierarchical model Odds ratios (89% CI)
Building: Intercept (Control, Instruction, Facilitator 1)	3.06 (1.99–4.61)	10.24 (7.23–14.76)	7.67 (1.95–18.89)
Caregiver: Intercept (Control, Instruction, Facilitator 1)	3.99 (3.00–5.31)	0.57 (0.35–0.93)	0.57 (0.17–1.41)
Patient: Intercept (Control, Instruction, Facilitator 1)	11.97 (9.22–15.41)	1.26 (0.81–1.97)	0.88 (0.16–2.38)
Patientpsych: Intercept (Control, Instruction, Facilitator 1)	1.60 (1.14–2.18)	0.51 (0.29–0.89)	0.42 (0.13–1.05)
SDOH: Intercept (Control, Instruction, Facilitator 1)	2.21 (1.43–3.39)	12.96 (9.08–18.54)	10.75 (3.04–26.13)
Canmeds: Intercept (Control, Instruction, Facilitator 1)	8.27 (6.23–10.70)	6.90 (4.84–9.95)	6.28 (1.41–17.74)
Expertise: Intercept (Control, Instruction, Facilitator 1)	4.43 (3.34–5.81)	1.37 (0.85–2.18)	0.80 (0.16–2.01)
Recommendations: Intercept (Control, Instruction, Facilitator 1)	2.08 (1.46–2.92)	0.98 (0.58–1.61)	0.78 (0.25–1.91)
Building by Condition (Intervention)		0.26 (0.17–0.38)	0.49 (0.18–1.92)
Building by Session Type (Initial, Homecare)		0.07 (0.03–0.16)	0.07 (0.03–0.16)
Building by Session Type (Initial, Debrief)		0.11 (0.06–0.18)	0.09 (0.05–0.15)
Building by Session Type (Follow-up, Homecare)		0.10 (0.04–0.22)	0.10 (0.04–0.23)
Building by Session Type (Follow-up, Debrief)		0.37 (0.21–0.65)	0.33 (0.18–0.60)
Building by Facilitator (2)		3.65 (2.46–5.42)	2.57 (1.31–4.87)
Building by Condition (Intervention) by Session Type (Initial, Homecare)		0.70 (0.20–2.07)	0.68 (0.20–2.11)
Building by Condition (Intervention) by Session Type (Initial, Debrief)		0.86 (0.49–1.51)	0.90 (0.50–1.61)
Building by Condition (Intervention) by Session Type (Follow-up, Homecare)		0.71 (0.27–1.77)	0.90 (0.33–2.31)
Building by Condition (Intervention) by Session Type (Follow-up, Homecare)		1.81 (0.90–3.58)	1.72 (0.85–3.45)
Building by Condition (Intervention) by Session Type (Initial, Homecare) by Facilitator (2)		0.90 (0.33–2.44)	0.76 (0.28–2.14)
Building by Condition (Intervention) by Session Type ((Initial, Debrief) by Facilitator (2)		2.36 (1.32–4.26)	2.74 (1.50–5.05)
Building by Condition (Intervention) by Session Type (Follow-up, Homecare) by Facilitator (2)		3.28 (1.40–8.16)	2.50 (1.03–6.28)
Building by Condition (Intervention) by Session Type (Follow-up, Homecare) by Facilitator (2)		0.84 (0.44–1.65)	0.87 (0.44–1.74)
Caregiver by Condition (Intervention)		2.14 (1.30–3.50)	1.61 (0.44–4.35)
Caregiver by Session Type (Initial, Homecare)		11.59 (6.20–22.19)	10.66 (5.58–20.37)
Caregiver by Session Type (Initial, Debrief)		6.40 (3.68–11.22)	6.03 (3.45–10.58)
Caregiver by Session Type (Follow-up, Homecare)		8.40 (4.20–16.68)	7.68 (3.88–15.36)
Caregiver by Session Type (Follow-up, Debrief)		14.31 (7.88–26.35)	14.24 (7.69–26.37)
Caregiver by Facilitator (2)		3.45 (2.11–5.70)	2.99 (1.60–5.44)
Caregiver by Condition (Intervention) by Session Type (Initial, Homecare)		0.32 (0.16–0.65)	0.34 (0.17–0.71)
Caregiver by Condition (Intervention) by Session Type ((Initial, Debrief)		0.13 (0.07–0.23)	0.13 (0.07–0.24)
Caregiver by Condition (Intervention) by Session Type (Follow-up, Homecare)		0.34 (0.15–0.78)	0.37 (0.16–0.86)
Caregiver by Condition (Intervention) by Session Type (Follow-up, Homecare)		0.87 (0.45–1.68)	0.93 (0.48–1.80)
Caregiver by Condition (Intervention) by Session Type (Initial, Homecare) by Facilitator (2)		0.53 (0.26–1.08)	0.60 (0.29–1.24)
Caregiver by Condition (Intervention) by Session Type ((Initial, Debrief) by Facilitator (2)		1.08 (0.59–1.97)	1.16 (0.63–2.11)
Caregiver by Condition (Intervention) by Session Type (Follow-up, Homecare) by Facilitator (2)		0.15 (0.06–0.35)	0.17 (0.07–0.41)
Caregiver by Condition (Intervention) by Session Type (Follow-up, Homecare) by Facilitator (2)		0.59 (0.31–1.12)	0.62 (0.32–1.21)
Patient by Condition (Intervention)		1.73 (1.09–2.75)	1.28 (0.35–4.01)
Patient by Session Type (Initial, Homecare)		32.12 (18.27–56.11)	30.77 (17.51–55.17)
Patient by Session Type (Initial, Debrief)		3.69 (2.22–6.10)	3.45 (2.09–5.72)
Patient by Session Type (Follow-up, Homecare)		21.74 (12.22–39.21)	21.47 (12.05–39.08)
Patient by Session Type (Follow-up, Debrief)		10.77 (6.22–19.36)	10.63 (6.05–18.78)
Patient by Facilitator (2)		2.44 (1.55–3.83)	2.21 (1.27–3.78)
Patient by Condition (Intervention) by Session Type (Initial, Homecare)		0.55 (0.30–1.02)	0.58 (0.31–1.10)
Patient by Condition (Intervention) by Session Type ((Initial, Debrief)		0.15 (0.09–0.26)	0.15 (0.09–0.26)
Patient by Condition (Intervention) by Session Type (Follow-up, Homecare)		0.89 (0.46–1.76)	0.94 (0.49–1.86)
Patient by Condition (Intervention) by Session Type (Follow-up, Homecare)		0.92 (0.50–1.72)	0.98 (0.53–1.82)
Patient by Condition (Intervention) by Session Type (Initial, Homecare) by Facilitator (2)		0.63 (0.34–1.18)	0.68 (0.36–1.30)
Patient by Condition (Intervention) by Session Type ((Initial, Debrief) by Facilitator (2)		2.31 (1.33–4.04)	2.46 (1.39–4.29)
Patient by Condition (Intervention) by Session Type (Follow-up, Homecare) by Facilitator (2)		0.88 (0.46–1.73)	0.95 (0.48–1.89)
Patient by Condition (Intervention) by Session Type (Follow-up, Homecare) by Facilitator (2)		1.03 (0.56–1.92)	1.11 (0.59–2.09)
Patientpsych by Condition (Intervention)		0.56 (0.29–1.06)	0.70 (0.25–2.28)
Patientpsych by Session Type (Initial, Homecare)		4.14 (1.93–8.74)	4.39 (2.03–9.39)
Patientpsych by Session Type (Initial, Debrief)		1.77 (0.91–3.46)	1.73 (0.90–3.40)
Patientpsych by Session Type (Followup, Homecare)		3.43 (1.56–7.42)	3.58 (1.61–7.81)
Patientpsych by Session Type (Followup, Debrief)		6.65 (3.37–13.25)	7.65 (3.78–15.69)
Patientpsych by Facilitator (2)		1.12 (0.59–2.07)	1.15 (0.55–2.37)
Patientpsych by Condition (Intervention) by Session Type (Initial, Homecare)		2.41 (1.03–5.77)	2.38 (1.01–5.72)
Patientpsych by Condition (Intervention) by Session Type ((Initial, Debrief)		0.91 (0.44–1.92)	0.93 (0.44–2.01)
Patientpsych by Condition (Intervention) by Session Type (Follow-up, Homecare)		2.22 (0.92–5.30)	2.14 (0.88–5.18)
Patientpsych by Condition (Intervention) by Session Type (Follow-up, Homecare)		1.59 (0.71–3.54)	1.51 (0.67–3.44)
Patientpsych by Condition (Intervention) by Session Type (Initial, Homecare) by Facilitator (2)		1.65 (0.72–3.86)	1.58 (0.67–3.80)
Patientpsych by Condition (Intervention) by Session Type ((Initial, Debrief) by Facilitator (2)		3.48 (1.65–7.43)	3.24 (1.52–7.07)
Patientpsych by Condition (Intervention) by Session Type (Follow-up, Homecare) by Facilitator (2)		3.66 (1.51–8.80)	3.85 (1.58–9.57)
Patientpsych by Condition (Intervention) by Session Type (Follow-up, Homecare) by Facilitator (2)		2.69 (1.21–6.03)	2.47 (1.10–5.61)
SDoH by Condition (Intervention)		0.22 (0.15–0.33)	0.40 (0.15–1.80)
SDoH by Session Type (Initial, Homecare)		0.08 (0.04–0.18)	0.08 (0.03–0.18)
SDoH by Session Type (Initial, Debrief)		0.05 (0.03–0.09)	0.04 (0.02–0.08)
SDoH by Session Type (Follow-up, Homecare)		0.05 (0.02–0.12)	0.05 (0.02–0.12)
SDoH by Session Type (Follow-up, Debrief)		0.06 (0.03–0.13)	0.06 (0.02–0.12)
SDoH by Facilitator (2)		1.95 (1.32–2.92)	1.78 (0.95–3.21)
SDoH by Condition (Intervention) by Session Type (Initial, Homecare)		3.88 (1.41–10.43)	3.25 (1.19–8.90)
SDoH by Condition (Intervention) by Session Type ((Initial, Debrief)		1.19 (0.58–2.40)	1.10 (0.53–2.26)
SDoH by Condition (Intervention) by Session Type (Follow-up, Homecare)		0.68 (0.16–2.65)	0.65 (0.15–2.48)
SDoH by Condition (Intervention) by Session Type (Follow-up, Homecare)		2.12 (0.72–5.82)	2.01 (0.67–5.66)
SDoH by Condition (Intervention) by Session Type (Initial, Homecare) by Facilitator (2)		0.20 (0.06–0.61)	0.20 (0.06–0.61)
SDoH by Condition (Intervention) by Session Type ((Initial, Debrief) by Facilitator (2)		1.49 (0.72–3.03)	1.50 (0.71–3.11)
SDoH by Condition (Intervention) by Session Type (Follow-up, Homecare) by Facilitator (2)		0.39 (0.11–1.32)	0.34 (0.09–1.14)
SDoH by Condition (Intervention) by Session Type (Follow-up, Homecare) by Facilitator (2)		0.69 (0.25–1.84)	0.67 (0.25–1.79)
Canmeds by Condition (Intervention)		4.31 (2.95–6.26)	1.99 (0.40–6.43)
Canmeds by Session Type (Initial, Homecare)		0.60 (0.33–1.09)	0.55 (0.30–1.01)
Canmeds by Session Type (Initial, Debrief)		0.32 (0.20–0.51)	0.30 (0.19–0.48)
Canmeds by Session Type (Follow-up, Homecare)		0.69 (0.37–1.26)	0.66 (0.36–1.22)
Canmeds by Session Type (Follow-up, Debrief)		1.59 (0.97–2.67)	1.45 (0.86–2.46)
Canmeds by Facilitator (2)		1.49 (1.03–2.18)	1.31 (0.82–2.06)
Canmeds by Condition (Intervention) by Session Type (Initial, Homecare)		0.33 (0.17–0.65)	0.33 (0.17–0.65)
Canmeds by Condition (Intervention) by Session Type ((Initial, Debrief)		0.09 (0.05–0.14)	0.08 (0.05–0.14)
Canmeds by Condition (Intervention) by Session Type (Follow-up, Homecare)		0.27 (0.13–0.55)	0.25 (0.12–0.51)
Canmeds by Condition (Intervention) by Session Type (Follow-up, Homecare)		0.19 (0.11–0.35)	0.19 (0.11–0.35)
Canmeds by Condition (Intervention) by Session Type (Initial, Homecare) by Facilitator (2)		1.13 (0.58–2.24)	1.31 (0.66–2.58)
Canmeds by Condition (Intervention) by Session Type ((Initial, Debrief) by Facilitator (2)		4.85 (2.93–8.21)	5.21 (3.11–8.71)
Canmeds by Condition (Intervention) by Session Type (Follow-up, Homecare) by Facilitator (2)		1.41 (0.72–2.88)	1.68 (0.82–3.48)
Canmeds by Condition (Intervention) by Session Type (Follow-up, Homecare) by Facilitator (2)		1.08 (0.59–1.93)	1.27 (0.70–2.36)
Expertise by Condition (Intervention)		0.29 (0.16–0.51)	0.48 (0.17–2.01)
Expertise by Session Type (Initial, Homecare)		5.22 (2.81–9.84)	5.64 (3.00–10.62)
Expertise by Session Type (Initial, Debrief)		1.68 (0.97–2.92)	1.76 (1.01–3.10)
Expertise by Session Type (Follow-up, Homecare)		8.56 (4.60–15.89)	9.02 (4.90–17.04)
Expertise by Session Type (Follow-up, Debrief)		4.53 (2.51–8.37)	5.05 (2.76–9.33)
Expertise by Facilitator (2)		0.82 (0.46–1.45)	0.91 (0.48–1.70)
Expertise by Condition (Intervention) by Session Type (Initial, Homecare)		4.32 (2.09–9.01)	4.45 (2.08–9.69)
Expertise by Condition (Intervention) by Session Type ((Initial, Debrief)		0.56 (0.29–1.10)	0.55 (0.27–1.12)
Expertise by Condition (Intervention) by Session Type (Follow-up, Homecare)		4.02 (1.87–8.76)	4.08 (1.87–9.05)
Expertise by Condition (Intervention) by Session Type (Follow-up, Homecare)		4.06 (1.98–8.51)	4.11 (1.94–8.73)
Expertise by Condition (Intervention) by Session Type (Initial, Homecare) by Facilitator (2)		3.93 (1.88–8.27)	3.55 (1.68–7.46)
Expertise by Condition (Intervention) by Session Type ((Initial, Debrief) by Facilitator (2)		8.70 (4.36–17.12)	7.79 (3.93–15.38)
Expertise by Condition (Intervention) by Session Type (Follow-up, Homecare) by Facilitator (2)		2.40 (1.13–5.10)	2.28 (1.07–4.99)
Expertise by Condition (Intervention) by Session Type (Follow-up, Homecare) by Facilitator (2)		4.12 (2.02–8.54)	3.68 (1.79–7.72)
Recommendations by Condition (Intervention)		0.45 (0.26–0.76)	0.61 (0.23–2.08)
Recommendations by Session Type (Initial, Homecare)		0.57 (0.22–1.37)	0.56 (0.22–1.38)
Recommendations by Session Type (Initial, Debrief)		2.15 (1.20–3.85)	2.22 (1.25–3.95)
Recommendations by Session Type (Follow-up, Homecare)		0.19 (0.06–0.57)	0.19 (0.06–0.58)
Recommendations by Session Type (Follow-up, Debrief)		2.40 (1.25–4.60)	2.58 (1.32–5.07)
Recommendations by Facilitator (2)		3.83 (2.28–6.64)	4.12 (2.16–7.77)
Recommendations by Condition (Intervention) by Session Type (Initial, Homecare)		1.75 (0.62–4.93)	1.89 (0.67–5.15)
Recommendations by Condition (Intervention) by Session Type ((Initial, Debrief)		0.71 (0.38–1.33)	0.73 (0.39–1.39)
Recommendations by Condition (Intervention) by Session Type (Follow-up, Homecare)		0.68 (0.16–2.74)	0.69 (0.16–2.74)
Recommendations by Condition (Intervention) by Session Type (Follow-up, Homecare)		5.55 (2.72–11.49)	5.97 (2.98–12.14)
Recommendations by Condition (Intervention) by Session Type (Initial, Homecare) by Facilitator (2)		0.62 (0.22–1.69)	0.59 (0.21–1.62)
Recommendations by Condition (Intervention) by Session Type ((Initial, Debrief) by Facilitator (2)		1.17 (0.61–2.22)	1.03 (0.54–1.95)
Recommendations by Condition (Intervention) by Session Type (Follow-up, Homecare) by Facilitator (2)		0.45 (0.12–1.62)	0.43 (0.11–1.59)
Recommendations by Condition (Intervention) by Session Type (Follow-up, Homecare) by Facilitator (2)		1.28 (0.63–2.60)	1.16 (0.56–2.39)
Random effects			
σ^2^	−0.00		0.00
τ00	−0.01		−0.01
ICC	0.03		−0.08
N	16 _Group_		16 _Group_ 2 _Condition_
Observations	3854	3854	3854

**Table:** How–regression summary table

Predictors	Intercept-only model Odds Ratios (89% CI)	Non-hierarchical model Odds Ratios (89% CI)	Hierarchical model Odds Ratios (89% CI)
Intercept (Control, Instruction)	0.14 (0.11–0.17)	0.29 (0.23–0.36)	0.51 (0.19–3.35)
Condition (Intervention)		1.56 (1.25–1.94)	1.41 (0.38–4.05)
Session Type: Initial, Homecare		0.20 (0.12–0.32)	0.22 (0.13–0.36)
Session Type: Initial, Debrief		0.32 (0.22–0.46)	0.31 (0.21–0.46)
Session Type: Follow-up, Homecare		0.05 (0.02–0.11)	0.06 (0.02–0.12)
Session Type: Follow-up, Debrief		0.23 (0.14–0.36)	0.23 (0.14–0.38)
Facilitator (2)		0.72 (0.58–0.89)	0.77 (0.51–1.18)
Condition (Intervention) by Session Type (Initial, Homecare)		0.41 (0.21–0.80)	0.35 (0.18–0.68)
Condition (Intervention) by Session Type (Initial, Debrief)		1.39 (0.95–2.04)	1.39 (0.93–2.05)
Condition (Intervention) by Session Type (Follow-up, Homecare)		0.41 (0.14–1.16)	0.38 (0.13–1.07)
Condition (Intervention) by Session Type (Follow-up, Debrief)		0.93 (0.57–1.50)	0.89 (0.54–1.47)
Session Type (Initial, Homecare) by Facilitator (2)		0.72 (0.36–1.38)	0.65 (0.33–1.25)
Session Type (Initial, Debrief) by Facilitator (2)		2.03 (1.39–3.00)	2.09 (1.43–3.09)
Session Type (Follow-up, Homecare) by Facilitator (2)		0.55 (0.18–1.59)	0.52 (0.17–1.47)
Session Type (Follow-up, Debrief) by Facilitator (2)		1.23 (0.76–1.99)	1.25 (0.76–2.03)
Random effects			
σ^2^	0.00		−0.04
τ00	0.10		0.15
ICC	0.04		−0.40
N	16 _Group_		16 Group 2 Condition
Observations	3854	3854	3854
Marginal R^2^ / Conditional R^2^	0.000 / 0.021	0.091	0.168 / 0.108
